# Esophageal Melanosis: An Unknown Entity

**DOI:** 10.7759/cureus.29064

**Published:** 2022-09-12

**Authors:** Ankush Agarwal S, Kothai Gnanamoorthy, Arun K, Aishwarya V Athani

**Affiliations:** 1 General Medicine, Sri Ramaswamy Memorial (SRM) Medical College Hospital and Research Centre, Chennai, IND; 2 General Medicine, Sri Ramaswamy Memorial (SRM) Medical College and Research Institute, Chennai, IND; 3 Radiology, Sri Ramaswamy Memorial (SRM) Medical College Hospital and Research Centre, Chennai, IND

**Keywords:** premalignant, anaemia, gerd, hematology, esophageal melanosis

## Abstract

Esophageal melanosis is the proliferation of melanocytes in the squamous epithelium of the esophagus and the accumulation of melanin in the walls of the esophagus. Normal esophageal mucosa does not contain melanocytes. It is a rare disease of the digestive system, and its significance has yet to be fully understood. Various studies have attributed it to gastroesophageal reflux disease, but hard evidence supporting such a claim is lacking. Some studies also point towards it being a pre-malignant condition, and further evaluation is warranted for earlier detection and treatment. We hereby present a case of chronic iron deficiency anemia incidentally found to have esophageal melanosis, confirmed with histopathological examination.

## Introduction

Esophageal melanosis is a finding seen due to melanocytic proliferation in the squamous epithelium of the esophagus and melanin accumulation in the walls of the esophagus [1]. Normal esophageal mucosa is devoid of melanocytes. It is a rare disease of the digestive system, and its significance has yet to be fully understood. It has been attributed to chronic esophagitis and to gastroesophageal reflux disease, but hard evidence supporting such a claim is lacking [2]. Some studies, such as the one conducted by Yokoyama et al., also point toward it being a pre-malignant condition [3]. Further evaluation is warranted for earlier detection and follow-up.

## Case presentation

A 53-year-old female presented to the general medicine outpatient department (OP) with complaints of dyspnoea on exertion and generalised myalgia for one month. She denied having orthopnoea or paroxysmal nocturnal dyspnoea. She had no history of bleeding manifestations, recent trauma, or blood loss. She denied having any history of cardiac, renal, or thyroid abnormalities, but she had a history of multiple blood transfusions along with iron supplementation two years ago for severe iron deficiency anaemia. Her menstrual cycles were regular 30-day cycles with three days of flow. No recent change in menstrual flow was noted by the patient. On examination, she was found to have pallor with a raised jugular venous pulse. Her vitals and systemic examination were found to be normal.

Her blood parameters showed a hemoglobin of 5.3 g/dl, a red blood cell count of 3.3 million/cumm, packed cell volume (PCV) of 20%, mean corpuscular volume (MCV) of 62 fl, mean corpuscular hemoglobin (MCH) of 16 pg, and mean corpuscular hemoglobin concentration (MCHC) of 26 g/dl. She had a platelet count of 4,98,100/cumm (Table 1). A peripheral smear showed severe microcytic hypochromic anaemia. An iron profile was done which showed serum iron of 9 mcg/dl, total iron-binding capacity (TIBC) of 424 mcg/dl, and a transferrin saturation of 2%. Serum lactate dehydrogenase (LDH) was 173 U/L (Table 1).

**Table 1:** Blood parameters PCV: packed cell volume, MCV: mean corpuscular volume, MCH: mean corpuscular hemoglobin, MCHC: mean corpuscular hemoglobin concentration, TIBC: total iron-binding capacity, LDH: lactate dehydrogenase.

Name of the investigation	Values obtained	Reference values
Hemoglobin	5.3 g/dl	12–15 g/dl
Red blood cell count	3.3 million/cumm	3.8–4.8 million/cumm
PCV	20%	36–46%
MCV	62fl	83–101 fl
MCH	16 pg	27–32 pg
MCHC	26 g/dl	31.5–34.5 g/dl
Platelet count	4,98,100/cumm	1,50,000–4,50,000/cumm
Serum iron	9 mcg/dl	70–180 mcg/dl
TIBC	424 mcg/dl	250–425 mcg/dl
Percentage transferrin saturation	2%	20–50%
Serum LDH	173 U/L	125–220 U/L

She was diagnosed with iron deficiency anaemia. The stool examination was normal. A blood transfusion was done and the patient was subjected to a bone marrow examination which showed a grade 0 of iron stores, following which iron supplementation with ferric carboxymaltose in injectable form was given and the patient improved symptomatically.

**Figure 1:**
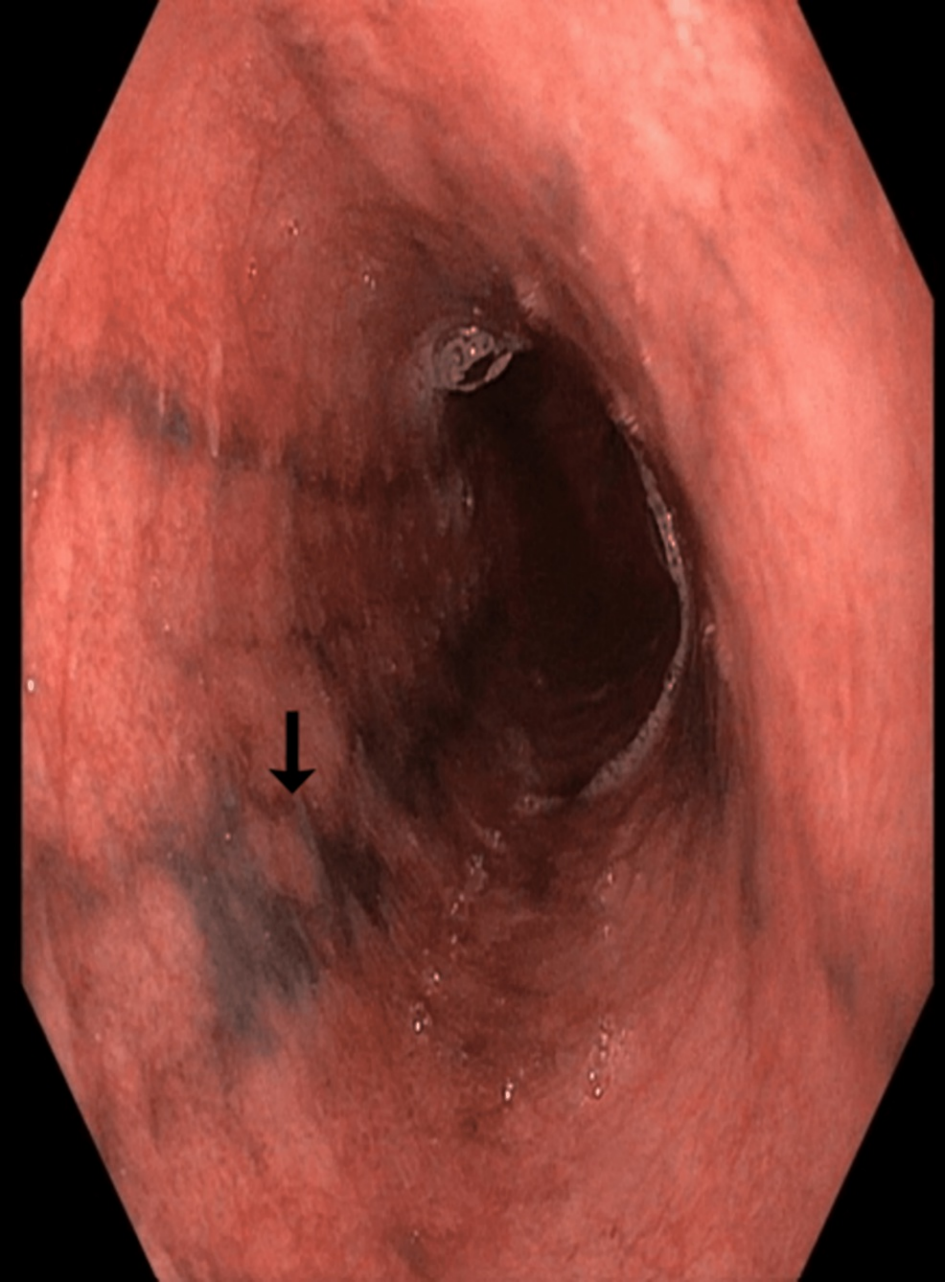
Upper gastrointestinal endoscopy image Darkly pigmented linear streaks seen in middle esophagus (black arrow).

**Figure 2:**
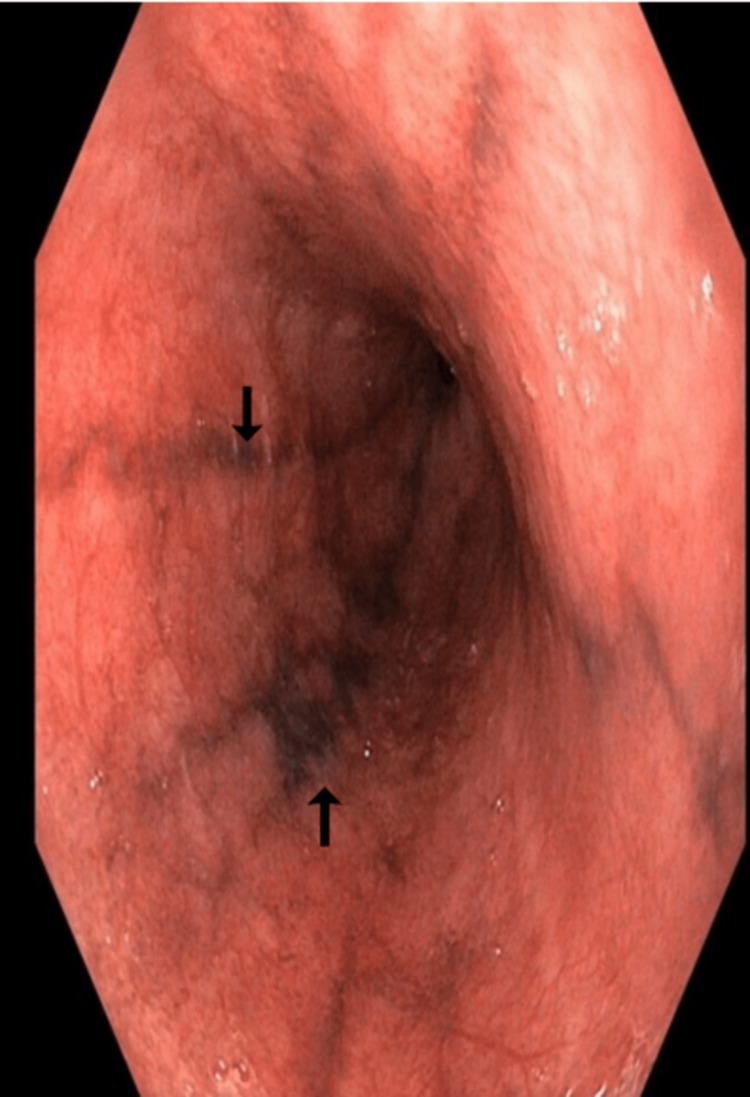
Upper gastrointestinal endoscopy image Darkly pigmented linear streaks in the middle and lower esophagus (black arrows).

She was subjected to upper gastrointestinal tract endoscopy as a part of further evaluation for anaemia, which showed darkly pigmented flat thin linear streaks in the mid and lower esophagus (Figures 1-2).

**Figure 3:**
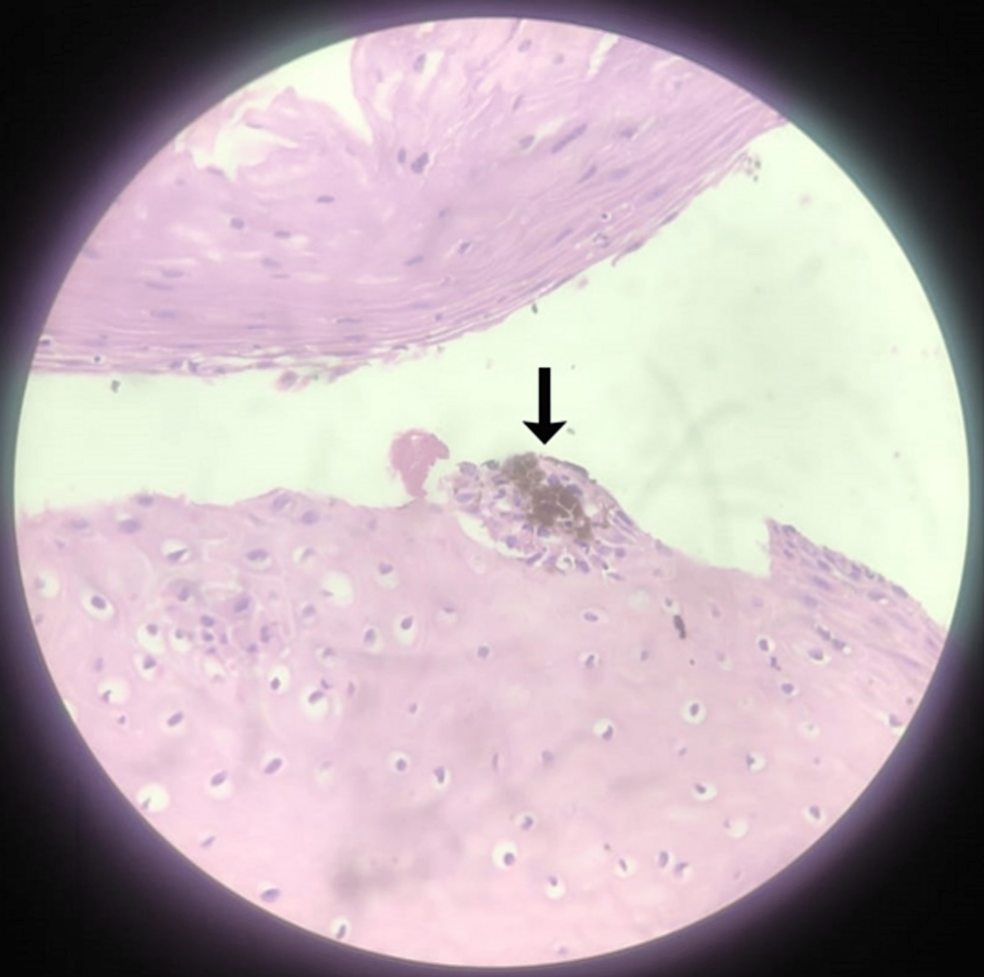
Biopsy image Forty times magnified image which shows the presence of intraepithelial melanophages (black arrow).

A biopsy was taken from the lesion and sent for histopathological examination, which revealed stratified squamous epithelium with intraepithelial melanophages (Figure 3).

**Figure 4:**
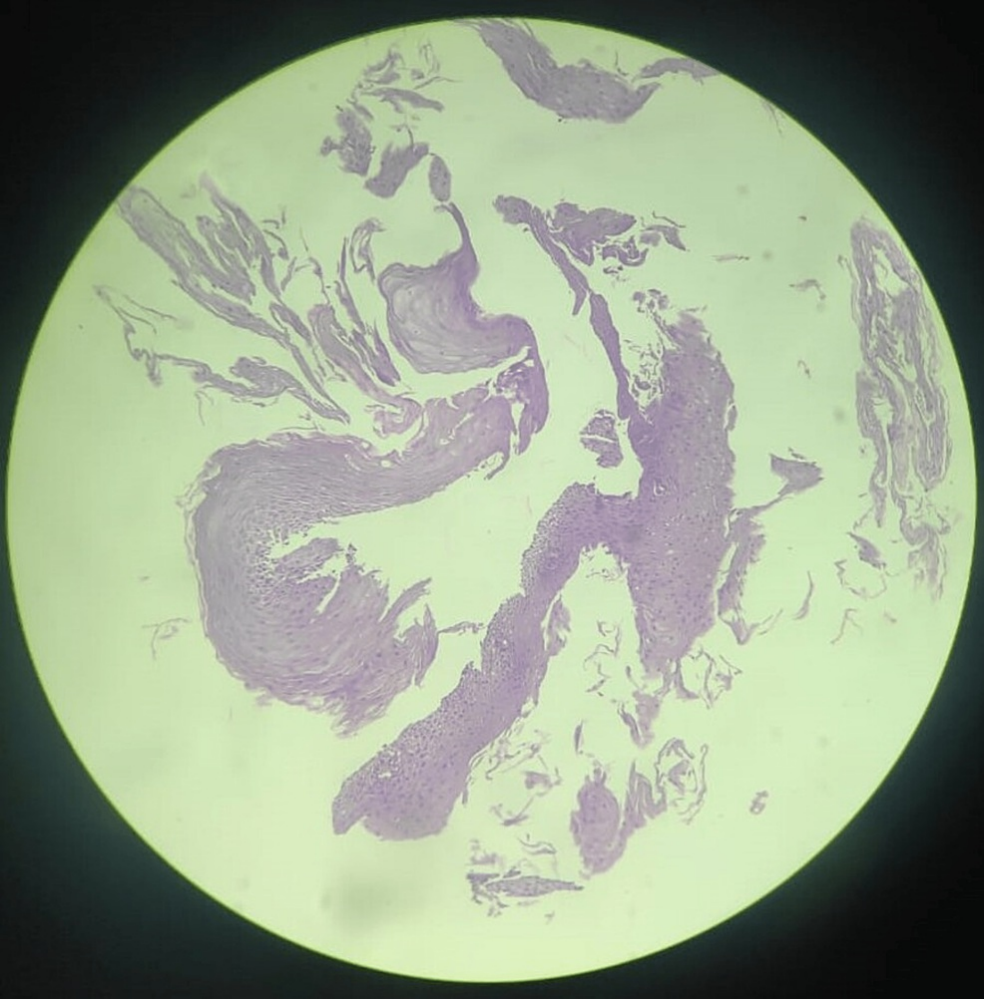
Biopsy image after melanin bleach procedure No pigmentation seen after the melanin bleach.

It is paramount to differentiate between melanin and hemosiderin in the biopsy sample, and this is usually done by the melanin bleach procedure. In our case, there was no black pigmentation following the procedure, suggesting that the pigment was melanin and had gotten bleached (Figure 4).

The above histopathological examination proves the presence of melanin and, hence, esophageal melanosis was confirmed.

## Discussion

Esophageal melanosis is a rare entity seen in seemingly benign conditions such as chronic esophagitis and gastroesophageal reflux disease, but it should be differentiated from other similar disorders that could result in rather dangerous outcomes. It has been seen in patients with chronic alcohol consumption by Yokoyama et al. and exposure to acetaldehyde in individuals with the aldehyde dehydrogenase 2 (ALDH2) genotype being the precipitating factor for melanosis has been suggested [3]. Yokoyama et al. also reported that esophageal melanosis is a precursor to esophageal carcinoma, melanoma, and esophageal dysplasia [3]. This makes it essential to identify this disorder and follow up with patients regularly for earlier detection of possible malignancies and their treatment.

Esophageal melanosis should be differentiated from disorders such as primary pigmented melanoma, which is a rare disease of the digestive tract originating from the mid and lower parts of the esophagus and can be differentiated by its polypoid appearance [4,5]. The diagnosis is made by the presence of junctional melanocytic activity on histologic evaluation of the esophageal mucosa [4,5].

The other rare differential to be considered is esophageal necrosis. Acute esophageal necrosis when compared to esophageal melanosis has a characteristic circumferential black discoloration with underlying friable hemorrhagic tissue and it has a sharp transition to normal-appearing mucosa at the gastroesophageal junction [6,7]. Ingestion of corrosive agents can cause sloughing of the esophageal mucosa and blackish discolouration of the esophageal wall, mimicking esophageal melanosis.

Benign differentials such as pseudomelanosis where there is tissue deposition of pseudomelanin, a ‘wear and tear’ substance derived from lysosomal degradation [8], and coal dust or carbon deposition are to be considered before a diagnosis of esophageal melanosis is made [9].

## Conclusions

This particular case was followed up for one year and no adverse events were noted. Esophageal melanosis is yet to be studied in detail and its significance in the diagnostic world is yet to be determined. Any patient with such a finding should be evaluated further to rule out the other differentials and should be followed up regularly to detect malignancy, if any, at earlier stages.
